# Mindful Self-Compassion Training Reduces Stress and Burnout Symptoms Among Practicing Psychologists: A Randomized Controlled Trial of a Brief Web-Based Intervention

**DOI:** 10.3389/fpsyg.2018.02340

**Published:** 2018-11-27

**Authors:** Terese Eriksson, Linnea Germundsjö, Elisabeth Åström, Michael Rönnlund

**Affiliations:** Department of Psychology, Umeå University, Umeå, Sweden

**Keywords:** self-compassion, stress, burnout, intervention, web-based

## Abstract

**Objective:** The aims of this study were (a) to examine the effects of a 6 weeks web-based mindful self-compassion program on stress and burnout symptoms in a group of practicing psychologists, and (b) to examine relationships between changes in self-compassion and self-coldness and changes in stress and burnout symptoms.

**Method:** In a randomized controlled trial, 101 practicing psychologists were assigned to a training group (*n* = 51) or a wait-list control group (*n* = 49). The training encompassed 15 min exercises per day, 6 days a week, for 6 weeks. The participants completed the Self-Compassion Scale (SCS), the Five Facets of Mindfulness Questionnaire (FFMQ), the Perceived Stress Scale (PSS), and the Shirom Melamed Burnout Questionnaire (SMBQ) pre and post intervention.

**Results:** Eighty-one participants (*n* = 40 in the training group, *n* = 41 in the control group) took part in the pre and post intervention assessments. Selective gains for the intervention group were observed for SCS total scores (*d* = 0.86; *d* = 0.94 for the SCS), FFMQ scores (*d* = 0.60), while levels of self-coldness was reduced (*d* = 0.73). Critically, levels of perceived stress (*d* = 0.59) and burnout symptoms (*d* = 0.44 for SMBQ total) were additionally lowered post intervention. Finally, the results confirmed the hypothesis that the measures of distress would be more strongly related to self-coldness than self-compassion, a pattern seen in cross-sectional analyses and, for burnout, also in the longitudinal analyses.

**Conclusions:** This training program appeared effective to increase self-compassion/reduce self-coldness, and to alleviate stress and symptoms of burnout and provide support of the distinction between self-compassion and self-coldness. Additional studies, preferably three-armed RCTs with long-term follow-up, are warranted to further evaluate the effectiveness of the program.

## Introduction

Both work-related stress and burnout are of serious concern from viewpoint of individual suffering as well as costs for the society. Particularly startling figures emanate from work sectors that may be considered as “emotionally draining,” such as healthcare. Research (e.g., Coyle et al., 2005; Morse et al., 2012) suggests that people in professions that require extensive education, such as social workers and priests, are at particular risk of stress and stress-related forms of mental ill-health. Stress and burnout among helping professionals is furthermore of concern in terms of how it might affect the quality of patient care. Studies have shown that burnout among staff is associated with lower patient satisfaction and longer recovery times (for a review, see Raab, 2014). Hence, finding ways to prevent mental ill-health and promote emotional resilience among helping professionals is an important issue, not only for the concerned individuals but also for those in need of care.

Work-stress and burnout have been regarded to reflect job characteristics (e.g., job demands and resources, Demerouti et al., 2001), but personal resources are in addition important to consider (e.g., Xanthopulou et al., 2007). In recent years, self-compassion has been identified as a personal resource that may be important to consider in relation to work-related stress (Neff, 2003; Raab, 2014). Compassion is regarded as sympathetic consciousness of others' distress together with a desire to alleviate it, which is assumed to be supported by compassion toward the self (Neff, 2003). As such, compassion should be distinguished from empathic distress, an empathic response toward suffering that, unlike compassion, tend to be self-focused and, when pro-longed, predispose of fatigue, sometimes referred to as “compassion fatigue,” which is better labeled as “empathy distress fatigue” (Klimecki and Singer, 2011). In fact, compassion and empathic distress seem to reflect partly different neural mechanisms (for an overview, see Singer and Klimecki, 2014).

Self-compassion, as defined by Neff (2003) and Neff and Dahm (2014), involves three elements: (1) self-kindness (vs. self-judgment), (2) common humanity (vs. isolation), and (3) mindfulness (vs. over-identification). Self-kindness entails being warm and understanding toward yourself when suffering. Common humanity involves recognition that suffering and personal inadequacy are part of human experience that is shared. Finally, mindfulness, broadly defined, involves “…paying attention in a particular way: on purpose, in the present moment, and non-judgmentally.” (Kabat-Zinn, 1994, p. 4) but, according to Neff and Germer (2013), mindfulness in the context of the self-compassion concept is more narrow, and refers to “the balanced awareness of negative thought and feelings involved in personal suffering” (p. 2).

To operationalize the self-compassion construct, Neff (2003) developed the Self-Compassion Scale (SCS), a self-report instrument extensively used in research on self-compassion. SCS consists of six subfactors (self-kindness, common humanity, mindfulness, self-judgment, isolation, and over-identification) that, when combined, measures an individual's total level of self-compassion. Degree of self-compassion as reflected by total score on the SCS has been found to be negatively associated with multiple forms of mental health (for a meta-analysis, see MacBeth and Gumley, 2012) and remains a negative predictor of anxiety and depression even when self-criticism and negative affect are adjusted for (Neff, 2003; Neff et al., 2017). In addition, research confirms the assumption that higher levels of self-compassion are associated with increased odds of showing helping intentions, such as lending one's mobile phone to a stranded stranger to let them call for help (Welp and Brown, 2013).

Importantly, scholars have suggested that self-compassion may be ameliorated by training and that such training is effective to reduce psychological distress (Raab, 2014). Self-compassion training might be especially relevant for workers in healthcare and other helping professionals, as it may facilitate the ability to cope with stress as well as boosts empathy (compassion) for patients. As stated by Raab (2014): “MBSR [mindfulness-based stress reduction] and self-compassion training are recommended for healthcare workers to decrease perceived stress and burnout and to increase self-compassion and empathy for patients” (p. 104).

A recent review of the literature of on self-compassion in healthcare providers (Sinclair et al., 2017a) identified 16 intervention studies targeting changes in self-compassion and other outcomes. However, four of the studies involved students (e.g., in nursing and medicine) rather than working professionals. Of primary concern, only one of the studies (Shapiro et al., 2005) was a randomized control (RCT) study (i.e., using random assignment to an intervention and control group; the other studies used a pre-repost design with no control group). The study by Shapiro et al. (2005) which involved a group of health care providers (nurses, social workers, psychologists) found an increase in self-compassion and a decrease in perceived stress following the intervention, but observed no changes in satisfaction in life nor symptoms of burnout following the intervention. In common with the majority of studies reviewed by Sinclair et al. (2017a), the training in Shapiro et al. involved MBSR, i.e., “standard” mindfulness training.

Whereas, as noted, mindfulness and self-compassion partly overlap conceptually, and self-compassion and mindfulness are commonly thought to be fostered by a non-judgmental attitude toward experience, recent years have seen the development of programs involving mindfulness but with a specific focus on self-compassion training (e.g., loving kindness meditation). Neff and Germer (2013) labeled their hybrid (mindfulness/self-compassion) program “mindful self-compassion.” In a pilot study (Neff and Germer, 2013), this program was evaluated in psychology trainees. Apart from gains in self-compassion, the training was found to boost multiple aspects of well-being (Neff and Germer, 2013). Other studies based on similar programs indicated improved body satisfaction in women (Albertson et al., 2014), and reduced depressive symptoms and improved metabolic outcomes in diabetes patients (Friis et al., 2016). Intervention studies with a similar focus on self-compassion in helping professionals with more work experience are sparse. However, dos Santos et al. (2016) reported significant decreases in perceived stress, burnout, depression, and trait anxiety in sample of nurses, technicians, and nursing assistants who went through a 6 weeks program on mindfulness and loving kindness meditation.

Provided that feasibility and effectiveness can be ascertained in randomized controlled studies, web-based training programs should be of interest. A web-based format offers several advantages over a face-to-face format, including fast and easy access, and hence, cost effectiveness compared with programs using a face-to-face format (e.g., Andersson and Cuijpers, 2009; Andersson and Titov, 2014). Whereas, the effectiveness of internet-delivered mindfulness-based interventions on various aspects of health has been demonstrated in a number of studies (e.g., Morledge et al., 2013; Boettcher et al., 2014; Henriksson et al., 2016; for a meta-analysis, see Spijkermann et al., 2016), the evidence with regard to programs that involve a special focus on self-compassion is still limited. A feasibility study by Krieger et al. (2016) demonstrated effects in a self-referred sample of participants suffering from harsh self-criticism. Finaly-Jones et al. (2017) moreover provided support of the effectiveness of a 6 weeks online program by showing reduced depressive symptoms, anxiety and stress, in parity with effects for face-to-face programs in a group of psychology trainees. Randomized controlled studies of internet-delivered programs targeting helping professionals, still seem to be lacking.

### The present study

Given the limited evidence of programs designed to improve self-compassion among helping professionals, in particular web-based interventions, the first objective of the present study was to evaluate the effects of a 6 weeks internet-delivered program designed to cultivate self-compassion skills and increase compassion toward others. The program involves exercises targeting mindfulness as well as self-compassion. Hence, in common with the program developed by Neff and Germer (2013), it may be considered to be a mindful self-compassion program. In regard to outcome measures, we paid attention to training-related changes in measures of mindfulness as well as self-compassion with level of perceived stress and burnout symptoms as the primary outcome measures, using an RCT design.

To evaluate the effectiveness of this program, we recruited a group of practicing psychologists. This was motivated by the fact that psychologists are a group of helping professionals for which high rates of stress-related conditions/disorders were observed in prior studies (Coyle et al., 2005; Morse et al., 2012). Indeed, a report from the Swedish Social Security Agency [Försäkringskassan] (2015) confirmed this pattern, and showed that as much of six percent of all Swedish psychologists were on sick leave 3 weeks or more related to stress, anxiety, or burnout during a working year; nearly twice the rate observed for the same group 8 years earlier.

A secondary aim was to examine subcomponents of the SCS related to the mental health factors (i.e., stress and burnout). More specifically, recent studies (e.g., López et al., 2015; Brenner et al., 2017) have suggested that the SCS does not reflect a unitary construct, but rather consists of two broad and theoretically distinct factors: self-compassion (self-kindness, common humanity, mindfulness scales combined) and self-coldness (i.e., self-judgment, isolation, and over-identification; for a review, see Brenner et al., 2018). Indeed, the two factors might be expected to be differentially related to well-being (positive affect) or psychological distress (negative affect), such that self-compassion is more closely related to well-being and positive mental health and self-coldness is more closely related mental ill-health (Brenner et al., 2018; Wadsworth et al., 2018). Hence, we aimed to examine whether changes in self-compassion and self-coldness had differential associations with stress and burnout respectively, and whether the patterns, so far only demonstrated for cross-sectional data, would generalize to longitudinal data. In line with prior research, we expected that self-coldness score would be more strongly associated than self-compassion score with measures of stress and burnout prior to the training. Based on the model by Brenner et al. (2018) we additionally predicted that changes in self-coldness would be more closely related to *changes* in stress and symptoms of burnout than to changes in the specific self-compassion component of the SCS.

## Materials and methods

### Participants

The study sample included 101 practicing psychologists, 97 women and 3 men (one participant providing a non-binary response to the question regarding gender). The participants ranged in age from 24 to 57 (*M* = 36.2, *SD* = 8.2). About half of the participants (49.5%) reported five or more than 5 years of work experience. Little more than a third (36.5%) were working in the psychiatry sector, 15.8% in primary health care, 13.9% work/organizations, 12.9% in schools, 3.0% in maternal health care. The remainder (17.8%) were in other work sectors. A majority of the participants (66.3%) reported very limited experience of mindfulness training (no training: 39.6%; on a few occasions: 26.7%) during the last 3 months, whereas the remainder (33.7%) reported having performed such exercises once a month or more often.

### Procedure

The participants were recruited via a closed Facebook group for psychologists/students of the psychologist program in Sweden. Inclusion criteria were: being employed at least at half time as a psychologist. Those willing to participate were randomly assigned to an intervention group (*n* = 52) and a wait-list control group (*n* = 49). The participants first completed a web-based survey created in Google Docs, including questions concerning basic demographic information, work experience, experience with mindfulness-based training (last 3 months; no training) and the battery of standardized questionnaires (see Instruments). The study protocols were approved by the regional ethics committee in Umeå and all participants provided informed consent prior to filling in the survey. Participants in the training group were thereafter provided unique log-in information required to start the training through e-mail. Seven weeks later, all participants were requested to respond to the questionnaires a second time. The participants were asked to indicate the number of steps in the program completed, data that could be verified electronically. Following completion of the second assessment, participants in the control group were provided free access to the online training program.

### Mindful self-compassion program

The training program (“Mindfulness and compassion with self and others”) was developed by Schenström (2017). In total, the 6 weeks program encompasses 10 h of training; about 15 min of training per day, 6 days a week. The program involves an initial instruction video that provides an outline of the program and informs of the procedure involved in using the program. The program is organized in six steps involving different types of exercises with guided instructions (auditory files). The stepwise organization of exercises means that a new exercise is available only once the prior exercise in the predetermined sequence of exercises has been completed. The steps in the program are labeled: (1) Kind attention, (2) Kind awareness, (3) Loving kindness with oneself and others, (4) Self-compassion—part 1, (5) Self-compassion—part 2, (6) Compassion with others and Quiet Practice. The program involves standard mindfulness exercises such as breathing anchor and body scans, and compassion-focused exercises such as loving-kindness, and exercises of compassion with self and others. An overview of the individual exercises included as part of each of the six steps of the program is provided in Table 1.

**Table 1:** Overview of exercises included in the six steps of the mindful self-compassion program.

**Exercise**	**Kind attention 1**	**Kind attention 2**	**Loving kindess—self and others**	**Self-compassion 1**	**Self-compassion 2**	**Compassion with others and quiet practice**
1	Breathing anchor	Explore breathing	Loving kindess with oneself	Compassion—close friend	Compassion—close friend	Compassion with self and others
2	Body scan	Mediation for thoughts and feelings	Body scan	Compassion—self	Compassion—self	Loving kindness and compassion
3	Explore breathing	Body scan	Loving kindness (self)	Compassion with self and others	Body scan	Quiet practice
4	Body scan	Breathing anchor	Loving kindness (self and others)	Pause for self-compassion	Pause for self-compassion	Pause for self-compassion
5	Breathing anchor	Mediation for thoughts and feelings	Loving kindness (self)	Compassion— self	Compassion— self	Loving kindness and compassion
6	Body scan	Body scan	Loving kindness (self and others)	Body scan	Explore breathing	Quiet Practice
7				Compassion with self and others	Compassion with self and others	Loving kindness and compassion
8				Pause for self-compassion	Pause for self-compassion	Quiet practice

Following completion of each of the exercises, instruction of an everyday exercise to be performed during the day is given (different exercises for the six steps). Participants can repeat exercises, and are presented with a graphical overview of completed steps and an overview of the forthcoming exercises within each step. The participants are also provided an online diary where their own reflections concerning the performed exercises may be registered.

### Self-compassion scale (SCS)

The SCS is a self-report instrument developed by Neff (2003) that contains 26 items. Each of the items is a statement (e.g., “When I'm going through a very hard time, I give myself the caring and tenderness I need.”, When I feel inadequate in some way, I try to remind myself that feelings of inadequacy are shared by most people.”), rated with regard to how often one reacts in the way described. The rating is made on a scale from *Almost never* (1) to *Nearly always* (5). The instrument was originally assumed to reflect six subscales: Self-kindness, self-judgment, common humanity, isolation, mindfulness and over-identification (Neff, 2003). In the present study, we considered the total (mean) score as devised by Neff (2003), justified by evidence of a single general self-compassion factor (e.g., Neff et al., 2017). Given arguments in other recent studies (e.g., López et al., 2015; Brenner et al., 2017) that the items rather reflect two broad factors: self-compassion (13 items) vs. self-coldness [13 items; labeled as “self-criticism” by López et al. (2015)], we additionally considered scores on the separate self-compassion and self-coldness scales. The internal consistency in the present sample (*n* = 101) was high both for the total scale (α = 0.92), the separate SCS (α = 0.88), and the self-coldness scale (α = 0.89).

### Five facets mindfulness questionnaire (FFMQ)

A Swedish version of the FFMQ (Lilja et al., 2011), originally developed by Baer et al. (2006), was used to assess mindfulness skills. Each of the items describe a particular state of mind (e.g., “I pay attention to sensations, such as the wind in my hair or sun on my face”) rated on a five-point Likert scale in regard to frequency of occurrence, from *Never/almost never* (1) to *Most of the time* (5). A sum score is usually taken to indicate the individuals' global level of mindfulness. The Swedish version of the questionnaire is slightly reduced and contains 29 of the 39 original items, with the same five facets as in the original questionnaire: (1) Non-reactivity to Inner Experience (six items), (2) Observing (noticing/attending to sensations/perceptions/thoughts/feelings; seven items), (3) Acting with Awareness (automatic pilot/concentration/non-distraction; five items), (4) Describing (labeling feelings/thoughts with words; six items), and Non-judging of Experience (five items). High levels of internal consistency were reported across the facets (α = 0.75–0.85) and for the total scale (α = 0.81; Lilja et al., 2011). Confirmatory factor analyses supported a hierarchical structure with four of the facets as first-order factors and a general mindfulness factor, but indicated that the facet Observe was not a significant part of a global structure (Lilja et al., 2011). Validity evidence include significant associations with meditation experience (Lilja et al., 2011), psychopathological symptoms and measures of well-being (Baer et al., 2008).

### Perceived stress scale (PSS)

This scale was developed by Cohen et al. (1983) and translated to Swedish by Eskin and Parr (1996). The 14 items describe experiences that life has been unpredictable, uncontrollable, or overly demanding during the last month (e.g., “In the last month, how often have you felt that you were unable to control the important things in your life”). Each of the described experiences are rated in regard to frequency of occurrence, from *Never* (0) to *Very often* (4). The ratings are summed across items (range: 0–56). High levels of internal consistency (α > 0.80) were observed and the evidence of criterion validity is well-documented (for a review, see Lee, 2012).

### Shirom-melamed burnout questionnaire (SMBQ)

Shirom Melamed Burnout Questionnaire (SMBQ) (Melamed et al., 1999). Shirom Melamed Burnout Questionnaire (SMBQ) consists of 22 questions involving burnout/exhaustion with four scales: “Physical Fatigue” (eight items, e.g., “I feel tired,” My batteries are dead), “Cognitive weariness” (six items, e.g., I have difficulty thinking of complex things), “Tension” (four items, e.g., “I feel tense”), and “Listlessness” (four items, e.g., “I feel full of vitality,” reverse coded). Five of the items are reverse coded. For each sub-domain, and the scale as a whole, the total score is averaged by dividing by the number of items in the domain. Items are rated on a seven-point Likert scale ranging from *Almost never* (1) to *Almost always* (7). The Swedish version (Lundgren-Nilsson et al. (2012) was found to exhibit adequate internal consistencies (α > 0.70) across the scales, but, based on Rasch-analyses, omission of the Tension factor was suggested (for an 18-item version; see Lundgren-Nilsson et al., 2012). Here, we used the 22-item version (computation of an 18-item score demonstrated virtually identical effects as those reported in the present study). Cutoff-values for the total mean score has been set to 3.75 (Grossi et al., 2003) or 4 (Stenlund et al., 2009).

### Statistical methods

The effects of the intervention were evaluated using mixed linear modeling in SPSS IBM 24. These analyses offers the advantage that an intention-to-treat approach can be used, such that all participants, whether measured both pre and post intervention or not, may be included, potentially yielding less biased effects than the frequently used mixed ANOVA model (e.g., Gueorguieva and Krystal, 2004). In these models, participant was a random effects factor, and time (pre-post) and group served as fixed factors. Covariance type used for the residuals, was set to be autoressive heterogenous. We tested full factorial models, i.e., effects of group, time and the interaction between the two factors, but were mainly interested in the interaction effects. Values for Cohen's *d* were computed according to the formula for pre- and post-test control group designs recommended by Morris (2008; dppc2—i.e., mean pre-post change in the treatment group minus the mean pre-post change in the control group, divided by the pooled pretest standard deviation). Analyses of simple correlations (Pearson's *r*) and hierarchical regressions were finally used to examine association of scores at baseline and association of change scores across the measures.

## Results

### Sample attrition

In the control group 41 out of the 49 participants completed the post-intervention measurements. In the treatment group, the corresponding figure was 40 out of 52 (i.e., 80.2%), 80 women and one man. The dropout rate did not differ between groups, χ^2^ (df = 1, *n* = 101) = 0.72, *p* = 0.40) and there was no indication of a difference between dropouts and returnees on any of the demographic variables or outcome measures. All of the participants in the intervention group had performed some training, but adherence varied from one step to complete adherence, a variable that was considered in subsequent analyses. Preliminary analyses were furthermore conducted to examine potential effects of prior training/experience with mindfulness. At this point, several alternative groupings of the participants were considered (see Participants) to form a dichotomized variable entered as a factor in the mixed linear models. The only significant effect observed, reflected the fact that participants reporting not having performed any kind of mindfulness training (last 3 months) scored significantly lower overall (i.e., across test occasions) on the measure of mindfulness skills (*M* = 3.17. *SE* = 0.074) than participants reporting some recent experience of mindfulness training prior to participating in the study (*M* = 3.38, *SE* = 0.059, *p* = 0.02). However, level of prior experience with mindfulness training did not interact with any of the other variables and was unrelated to the other outcome measures, suggesting the prior practices in mindfulness had no or only minor influence on the results of this study (i.e., in regard to the magnitude of the effects of the intervention on self-compassion, stress, and burnout symptoms).

### Pre- and post-intervention data

A summary of the means and standard deviations for the measures of self-compassion, mindfulness, perceived stress and burnout (SMBQ total scale and the four subscales) before and after the intervention is provided in Table 2.

**Table 2:** Pre-post-intervention scores (means and standard deviations) on the outcome measures in the intervention group and the control group.

	**Intervention group**						**Control group**							
	**Pre (all**, ***n*** = **52)**		**Pre (*****r*****;** ***n*** = **40)**		**Post (*****n*** = **40)**		**Pre (all**, ***n*** = **49)**		**Pre (*****r*****;** ***n*** = **41)**		**Post (*****n*** = **41)**		**G × T**	
**Outcome**	***M***	***SD***	***M***	***SD***	***M***	***SD***	***M***	***SD***	***M***	***SD***	***M***	***SD***	***F***	***d***
Self-com (SCS tot)	2.97	0.66	2.99	0.61	3.52	0.64	3.13	0.60	3.12	0.62	3.11	0.70	30.79^***^	0.86
- Self-compass	3.22	0.68	3.25	0.63	3.73	0.57	3.39	0.58	3.40	0.60	3.32	0.66	26.84^**^	0.94
- Self-coldness	3.29	0.75	3.26	0.72	2.69	0.76	3.14	0.75	3.15	0.76	3.11	0.85	22.90^***^	0.73
Mindfulness	3.14	0.46	3.13	0.45	3.51	0.44	3.23	0.53	3.24	0.56	3.32	0.54	19.12^***^	0.60
Perceived stress	25.38	25.38	25.28	6.99	19.43	6.42	25.12	8.02	25.22	8.44	23.49	8.56	11.64^***^	0.59
Burnout (SMBQ-t)	3.70	1.03	3.65	0.98	3.08	0.93	3.65	1.15	3.69	1.19	3.60	1.12	9.87^**^	0.44
- Physical Fatigue	3.67	1.16	3.66	1.14	3.08	1.10	3.70	1.39	3.79	1.45	3.35	1.31	5.54^*^	0.31
- Cognitive Weariness	3.36	1.34	3.30	1.39	2.70	1.38	3.42	1.41	3.30	1.33	3.32	1.24	8.15^**^	0.46
- Tension	3.98	1.26	3.86	1.23	3.24	1.02	3.71	1.12	3.79	1.15	3.56	1.27	4.53^*^	0.33
- Listlessness	4.00	1.34	3.98	1.12	3.52	1.08	3.85	1.38	3.96	1.40	4.01	1.34	6.73^*^	0.40

*F-values for the group-by-time (G × T) interaction and estimates of effect size (Cohen's d) are provided (rightmost columns)*.

* *p < .05*,

** *p < .01*,

*** *p < .001, SCS tot = Self-Compassion Scale, total score; SMBQ-t = Shirom-Melamed Burnout Questionnaire total score*.

Given our intention-to-treat (MLM) approach involving also those who failed to return for the post assessment, pre assessment data are presented both for the entire group and for the returnees. As can be seen, the groups of returnees show highly similar values as the whole group prior to the training/waiting period, which, as noted, reflected no apparent selectivity of dropout in regard to the measures in focus. Inspection of the mean values across measures for pre vs. post reveal a pattern of mean changes in the intervention group, but no or only minor changes in the control group. Consistent with this description, the mixed linear models revealed significant Group-by-Time interactions, throughout (see Table 2; *F*-values). Still, it is clear from the *F*-values and values for Cohen's *d* that the magnitude of effects varied across the measures. More specifically, the largest effect were seen for the SCS total score and the separate self-compassion score (composite of 13 items), effects that were large (*d* > 0.8, Cohen, 1988) with intermediate effect sizes (*d* > 0.50 and < 0.80; Cohen, 1988), in the upper range, for self-coldness and mindfulness (FFMQ) scores. Finally, the effects for the global burnout measure (SMBQ total) as well as the separate subscales were small (< 0.50; Cohen, 1988) and smallest for the Physical Fatigue scale (*d* = 0.31) and the Tension scale (*d* = 0.33).

### Associations of SCS components and distress

The foregoing analyses demonstrated substantial training-related gains in self-compassion and reductions of stress at the group level. Further support of the internal validity should be obtained if changes in the targeted construct (self-compassion) are associated also with degree of changes on the primary outcome measures, i.e., perceived stress and burnout symptoms. As stated in the introduction, we additionally set out to test specific hypothesis regarding relations of the latter measures to separate self-compassion and self-coldness scales. In these analyses we tested if (1) self-coldness, as we expected, would show a stronger association with perceived stress and burnout in cross-sectional analyses (i.e., prior to training) and, (2) changes in self-coldness, in accord with our prediction, would be more strongly associated also with changes in the two measures of distress than intervention-related changes in self-compassion. To investigate covariation of changes in the measures at the individual level, we computed change across the measures by regressing the pre-intervention score on the post-intervention score (i.e., so as to obtain residualized changes scores). The change scores were next submitted to a correlational analyses. The results of the correlational analyses are presented in Table 3. Correlations of the measures prior to training (cross-sectional data) are displayed on the left half of the table and correlations of change scores to the right.

**Table 3:** Correlations of SCS (total, self-compassion, self-coldness) scores and measures of distress at cross-sectional analyses (baseline scores) and association of change scores (longitudinal associations) of the corresponding measures.

	**Baseline scores (*****n*** = **101)**			**Change scores (*****n*** = **40)**		
**Variable**	**SCS-total**	**Self-comp**	**Self-cold**	**SCS-total**	**Self-comp**	**Self-cold**
Perceived stress (PSS)	−0.61^**^	−0.50^**^	0.61^**^^a^	−0.64^**^	−0.61^**^	0.59^**^
Burnout (SMBQ-tot)	−0.49^**^	−0.37^**^	0.52^**^^a^	−0.47^**^	−0.30	0.56^**^^a^
- Physical fatigue	−0.39^**^	−0.32^**^	0.38^**^	−0.29	−0.18	0.38^*^^b^
- Cognitive weariness	−0.41^**^	−0.26^**^	0.47^**^^a^	−0.50^**^	−0.28	0.61^**^^a^
- Tension	−0.55^**^	−0.42^**^	0.58^**^^a^	−0.44^**^	−0.33^*^	0.52^**^^b^
- Listlessness	−0.60^**^	−0.51^**^	0.57^**^	−0.55^**^	−0.47^**^	0.52^**^

* *p < 0.05*,

** *p < 0.01*.

a *significant difference in magnitude of correlation for self-compassion and self-coldness (reverse coded items) as determined by a z-test (p < 0.05)*.

b *p < 0.07*.

Beginning with the aggregate self-compassion, SCS total score was negatively associated with stress and burnout at baseline. In addition, changes in SCS total score were negatively associated with both global measure of distress and scores on the separate SMBQ subscales (*p* < 0.05), except Physical Fatigue, which showed a tendency in the same direction (*p* = 0.06).

The separation of SCS into self-compassion and self-coldness scores were generally supportive of our hypotheses. More specifically, self-coldness showed a significantly larger association with SMBQ total score than self-compassion, both in terms of baseline scores and change scores. A corresponding pattern was evident for three out of the four SMBQ subscales. Also, the measure of perceived stress (PSS) was significantly more closely associated with self-coldness (*r* = 0.61) than to self-compassion score (*r* = –0.50), but the expected pattern for change scores was not observed. Controlling for adherence (i.e., number of completed steps in the program by calculation of partial correlations), made little difference, suggesting that the associations were not simply reflective of this factor. Simple correlations of adherence with the change scores across the outcome measures, revealed a significant association of adherence with SCS (*r* = 0.27, *p* < 0.05, one-tailed), for PSS (*r* = −0.31, *p* < 0.05), though, with a tendency for SMBQ-total (*r* = −0.23, *p* = 0.07), but not for FFMQ score (*r* = 0.11, *p* = 0.24).

Finally, to determine whether improvements in mindfulness skills made a significant contribution to changes in stress and burnout symptoms, once changes in self-compassion were accounted for, we performed hierarchical regression analyses. Change score of each measure of distress (PSS, SMBQ-total) was the criterion, and residualized change scores in SCS total score (step 1) and change scores in FFMQ score (step 2) were used as the predictors. For intervention-related changes in PSS, SCS change score (β = −0.64, cf. Table 2) predicted 41.5% of the variance in step 1. In step 2, FFMQ change score accounted for 16.1% additional variance [*F*_change__(1, 37)_ = 14.04, *p* < 0.001], yielding a total *R*^2^ of 0.576 for the model. For SMBQ change score, SCS change entered in step 1, accounted for 29% of the variance (β = −0.467, *p* < 0.001). However, in step 2, FFMQ score added no significant variance [Δ*R*^2^ = 0.031, *F*_change__(1, 37)_ = 1.51, *p* = 0.23], total *R*^2^ = 0.249.

## Discussion

The objectives of this study were to examine the effects of a 6 weeks web-based mindful self-compassion program on stress and burnout symptoms in a sample of practicing psychologists, and to examine relationships between changes in the two putative components of the SCS (self-compassion and self-coldness) with stress and burnout symptoms. Beginning with the first aim, the results were, in several respects, supportive of the effectiveness of the intervention. First, the training increased self-compassion as reflected by the total SCS score and the separate SCS (excluding reverse coded items that may be regarded to reflect self-coldness). This is in line with prior studies involving compassion focused interventions with face-to-face format (Neff and Germer, 2013; Beaumont et al., 2016; but see dos Santos et al., 2016). The training group furthermore showed substantially improved mindfulness skills (see also Neff and Germer, 2013). These desired training outcomes were accompanied by significant reductions in perceived stress and burnout symptoms, with estimates of effect sizes comparable to those for similar interventions that used a face-to-face format for stress (e.g., Neff and Germer, 2013; dos Santos et al., 2016) and the global measure of burnout (dos Santos et al., 2016). A more detailed analysis of the four SMBQ subscales reflecting aspects of burnout, were suggestive of slightly larger effects for the two scales emphasizing mental symptoms (Cognitive Weariness, Listlessness) compared with the two scales involving physical symptoms (Physical Fatigue, Tension), a finding which appears reasonable given the present focus of the training.

Overall, the effect sizes in the present study appeared comparable to those obtained in the few similar studies using a face-to-face format. This is promising to the extent that internet-delivered training, as noted, have a number of advantages. More specifically, internet-delivered training may have the advantages of (1) easy access, without waiting lists; (2) availability in the home environment, which saves traveling time and enable people to work at their own pace; (3) permission for users to remain anonymous without a need to adopt a patient role; (4) lack of requirement to involve a therapist educated in mindfulness; and (5) cost-effectiveness (see Andersson and Cuijpers, 2009; Andersson and Titov, 2014). Notwithstanding these advantages, there may be a few disadvantages of internet-delivered training as well. Andersson and Titov (2014) mentioned a few factors (in the context of CBT studies) that would seem to be of more general concern, including negative client and clinician attitudes toward interventions, the fact that clinicians may feel threatened if internet delivered interventions are disseminated. Potential threats to integrity associated with less than perfect security of internet-delivered information may furthermore be of concern. There is also the risk that individuals who suffer from more serious disorders seek this type of internet-delivered training rather than some more appropriate form of treatment. Future studies will have to evaluate such pros and cons of web-based training to comparisons of face-to-face formats to draw any firm conclusions in regard to the relative merits of the current form of the program in regard to effectiveness.

The fact that the present training program had a larger effect on the measure of self-compassion than on the measure of mindfulness (large vs. intermediate effect) appears reasonable given the program's special focus on self-compassion (see also Neff and Germer, 2013). Also, the gains on the specific self-compassion dimension of the SCS was slightly larger than the corresponding reduction in score on the self-coldness dimension. Presumably, the relative effect of training on the subcomponents might vary depending on what groups of participants are considered; individuals suffering from particular high levels of self-criticism might, for example, show a reversed pattern (e.g., Wadsworth et al., 2018). Another explanation is that the intervention used in this study more closely operated to increase self-compassion than to decrease self-coldness, highlighting the need to customize self-compassion interventions according to the treatment goals. As pointed out by Brenner et al. (2018): “increasing self-compassion and decreasing self-coldness may be important goals to increase well-being, but specifically focusing on reducing self-coldness may be key in trying to reduce distress” (Brenner et al., 2018, p. 353). Indeed, more detailed analyses in the present study, provided partial support of the hypothesis based on prior evidence (e.g., Körner et al., 2015; Brenner et al., 2018) that measures of self-compassion and self-coldness exhibit different strength of associations with measures of distress. More specifically, in line with Brenner et al. (2018), self-coldness was more strongly associated with the measure of stress than self-compassion, but also with total burnout score and with three out of four SMBQ subscales in our cross-sectional analyses. Interestingly, the intervention-related changes in burnout were more closely associated with changes in self-coldness than with changes in self-compassion consistent with our second hypothesis (see Wadsworth et al., 2018 for a similar result in regard to anxiety and depression). Of further interest, training-related gains in mindfulness skills accounted for significant variance over and beyond changes in self-compassion in changes of perceived stress. Thus, despite substantial overlap likely attributable to a common core of the constructs (non-judgment of experience), self-compassion and mindfulness may account for unique variance in certain measures of distress (see also Neff and Germer, 2013). Finally, the fact that adherence to the training was significantly related to gains in self-compassion and reductions in stress, with a similar tendency for burnout, appears reasonable. No corresponding association of training dose and amount of gain was observed for the measure of mindfulness, which could reflect the fact that the mindfulness training was mainly concentrated at the early stages of the program.

Even though strengths of the study, including random assignment of participants and inclusion of a sample of working professionals (many prior studies involved student samples), might deserve to be pointed out, it clearly has weaknesses *that should be addressed in follow-up studies*. First, in order to isolate effects of self-compassion, future studies should consider inclusion of an active control group, i.e., a condition involving some alternative treatment, which should provide a better control for expectancy (placebo) effects. For this purpose, a program restricted to standard mindfulness training, matched with the self-compassion focused program in regard to duration and time requirements, should be of interest and should be valuable to sort out the unique aspects of the self-compassion component. Inclusion of measures of stress other than self-report measures, for example cortisol, should additionally be valuable to control for expectancy effects. Furthermore, even though Neff and Germer (2013) indicated good maintenance of the effects of a similar training program up to a year after completion of the program, the present study lacked a long-term follow-up. Future studies targeting helping professionals should furthermore benefit from measuring compassion for others, an aspect missing in most studies including the present one, although the extant measures have been criticized for having questionable validity and/or inadequate psychometric properties (Strauss et al., 2016; Sinclair et al., 2017b) and might therefore need to be further refined. A third limitation is that the overwhelming majority of the study participants were female. The gender biased sample of the present study probably reflects that the psychology profession is, in Sweden, more often occupied by females; that women to a higher degree experience burnout (Norlund et al., 2010); and finally, that women are more prone to help-seeking behaviors and attitudes (Wendt and Shafer, 2016). Nonetheless, the uneven gender distribution in the present study could suggest that the results are more applicable to women than to men. To our knowledge though, there are no empirical studies implying that effectiveness of self-compassion training is dependent on gender. Future studies should, finally, consider effectiveness in terms of improvement in patient outcomes following the training (Raab, 2014).

In conclusion, this study provided support of the effectiveness of the brief web-based mindful-self compassion intervention to increase self-compassion and reduce self-coldness, so as to lower levels of perceived stress and burnout in a sample of practicing psychologists. The fact that the present study was a randomized controlled study should give particular weight to the findings. Thus, even though more rigorous studies involving an active control group and long-term follow-up of the effects are warranted, the present study supports the suggestion (e.g., Egan et al., 2017) that interventions aimed to improve self-compassion may be an important means to prevent and reduce fatigue, stress and burnout among helping professionals.

## Author contributions

TE, LG, EÅ, and MR designed the study. MR performed the statistical analyses. TE and LG wrote the first draft. TE, LG, EÅ, and MR were involved in revising the manuscript.

### Conflict of interest statement

The authors declare that the research was conducted in the absence of any commercial or financial relationships that could be construed as a potential conflict of interest.
